# Exploration of population and practice characteristics explaining differences between practices in the proportion of hospital admissions that are emergencies

**DOI:** 10.1186/1471-2296-15-101

**Published:** 2014-05-21

**Authors:** Chantelle Elizabeth Wiseman, Richard Baker

**Affiliations:** 1 FY1 in ITU at Nevill Hall hospital, Cardiff, Wales, UK; 2 Department of Health Sciences, University of Leicester, 22-28 Princess Rd West, LE1 6TP, Leicester, UK

**Keywords:** Primary care, Health services, Health planning

## Abstract

**Background:**

Emergency (unscheduled) and elective (scheduled) use of secondary care varies between practices. Past studies have described factors associated with the number of emergency admissions; however, high quality care of chronic conditions, which might include increased specialist referrals, could be followed by reduced unscheduled care. We sought to characterise practices according to the proportion of total hospital admissions that were emergency admissions, and identify predictors of this proportion.

**Method:**

The study included 229 general practices in Leicestershire, Northamptonshire and Rutland, England. Publicly available data were obtained on scheduled and unscheduled secondary care usage, and on practice and patient characteristics: age; gender; list size; observed prevalence, expected prevalence and the prevalence gap of coronary heart disease, hypertension and stroke; deprivation; headcount number of GPs per 1000 patients; total and clinical quality and outcomes framework (QOF) scores; ethnicity; proportion of patients seen within two days by a GP; proportion able to see their preferred GP. Using the proportion of admissions that were emergency admissions, seven categories of practices were created, and a regression analysis was undertaken to identify predictors of the proportion.

**Results:**

In univariate analysis, practices with higher proportions of admissions that were emergencies tended to have fewer older patients, higher proportions of male patients, fewer white patients, greater levels of deprivation, smaller list sizes, lower recorded prevalence of coronary heart disease and stroke, a bigger gap between the expected and recorded levels of stroke, and lower proportions of total and clinical QOF points achieved. In the multivariate regression, higher deprivation, fewer white patients, more male patients, lower recorded prevalence of hypertension, more outpatient appointments, and smaller practice list size were associated with higher proportions of total admissions being emergencies.

**Conclusion:**

In monitoring use of secondary care services, the role of population characteristics in determining levels of use is important, but so too is the ability of practices to meet the demands for care that face them. The level of resources, and the way in which available resources are used, are likely to be key in determining whether a practice is able to meet the health care needs of its patients.

## Background

After several years of steady growth in the funding of health care, many European countries, including Britain, are now experiencing a decline in funding [1]. During the period of economic growth, investment in secondary care (specialists and hospital services) increased [1,2], but now approaches are needed to contain costs and improve efficiency. Primary care has a role to play by limiting the numbers of patients who have to be cared for in costly specialist settings. The delegation to primary care physicians of decisions about the commissioning of secondary care may encourage them to design services and care pathways that increase the proportion of patients who are managed in lower cost settings, and consequently clinical commissioning groups led by general practitioners are being introduced in England [3]. General practitioners are the primary care physicians in England, and they work in general practices that serve registered patient populations; every general practice is a member of a clinical commissioning group. Improved management of people with chronic conditions in primary care may help to avoid some emergency admissions [4,5]; management includes early intervention before urgent problems arise, and such interventions could increase the scheduled use of hospital services. However, although improved disease management may have potential to affect the proportion of admissions that are emergencies, it is also possible that some population characteristics, for example deprivation, promote both high scheduled and unscheduled use of hospital services.

Population factors (deprivation, age, ethnicity, gender) and practice factors (size, access and continuity of care, distance from hospital) have been shown to influence use of secondary care [6-11]. Service characteristics such as the numbers of specialists or referral pathways may also affect use. In order to help practices reduce the demand for secondary care, both practices and commissioning groups need to understand the needs of practice populations for health care, and the capacity of practices to meet a greater proportion of those needs. Practice profiles have been made available [12], containing information on local demography; Quality and Outcomes Framework (QOF) domains (a pay for performance scheme used in the UK to incentivise aspects of care) [13]; disease prevalence estimates; admission rates; and patient satisfaction. The data are available by practice, for all practices in a clinical commissioning group, and for groups of practices classified as being similar through having populations with similar levels of deprivation, or by reference to a set of characteristics used to define practice peer groups. The peer groups were identified through examination of the characteristics of age, gender, ethnicity and deprivation of the practice population, and location of the practice (urban, town, village) [14]. In a search of the relevant literature, we were able to identify only a few studies of methods to group, or type, practices. A typology of six practice types was developed to describe practices in Canada [15], and a taxonomy of primary care organizations in Canada is being developed in a study to track the evolution of primary care during a process of reform [16]. A taxonomy of a sample of Australian general practitioners concentrated on features of the practitioner and the health care team [17]. These classifications did not concentrate on variations in use of secondary care and are not suited to helping practices and or health systems identify and address the needs of practice populations [18].

The aim of our study was to characterise practices according to the proportion of total admissions that were emergencies, and to investigate whether the proportion is explained by characteristics of practice populations or by features of systematic management of chronic conditions by practices. We wished to explore whether a characterisation or typology based on the proportion of admissions that are emergencies might have potential in highlighting factors that could help commissioning groups and practices plan initiatives to contain use of secondary care.

## Methods

### Setting

The study took place in the English counties of Leicestershire, Northamptonshire and Rutland, where primary care services were administered by two primary care trusts until April 2013, when they were replaced by five clinical commissioning groups. There were three acute hospitals for medical and surgical conditions, two mental health hospitals, and several small community hospitals. Approximately 40% of the population of Leicester city are of an ethnic minority, predominantly South Asian [19].

### Secondary care use

For this study we used publically available data from the Health and Social Care Information Centre’s Indicator Portal, for the years 2010–2011 [20]. Information on the secondary care use of general practices included scheduled care – the number of outpatient appointments per 1000 patients and the number of elective admissions per 1000 patients, and unscheduled care - the number of emergency department attendances per 1000 patients and the number of emergency admissions per 1000 patients. We calculated the proportion of total admissions that were emergency admissions, and then assigned the practices to seven groups according to whether this proportion was up to 34.9%, 35.0-39.9%, 40.0-44.9%, 45.0-49.9%, 50.0-54.9, 55.0-59.9%, or 60.0% and over.

### Practice and patient population characteristics

We sought to describe the seven groups of practices with different proportions of admissions that were emergency according to characteristics of the practice populations, and characteristics of the practices themselves. The population characteristics were: proportion of patients aged 65 and over; gender; deprivation (the index of multiple deprivation or IMD, estimated using the average of the IMD scores for each lower super output area in which a given practice has registrations, weighted by the percentage of the practice’s registrations in each LSOA [20]); ethnicity; prevalence, expected prevalence and the difference between the expected and recorded prevalence (the prevalence gap) of coronary heart disease, hypertension and stroke. For practice characteristics we used: total and clinical quality and outcomes framework (QOF) scores for the 2010/11 year; proportion of patients seen by a GP within two working days; the proportion of patients able to see their preferred doctor; list size; and the headcount number of GPs per 1000 patients, a figure that excludes trainees and retained doctors (doctors contracted in order to maintain their clinical skills but who undertake few clinical sessions) [20].

Older age has been linked to greater numbers of emergency and elective hospital admissions, and gender has been associated with hospital admissions [8,21]. Lower practice list size and white ethnicity have been associated with emergency department attendance rates [9], and elective and emergency admissions [6]. Larger practices may be able to offer a wider range of services to people with chronic conditions, thereby reducing the need for hospital care [7]. We used the prevalence of coronary heart disease, hypertension and stroke as an estimate of the levels of chronic disease in each practice; this is because a greater burden of ill-health in the practice should lead to an increase in secondary care usage. The expected prevalence and prevalence gap also estimate whether practices are identifying the predicted amount of disease for their patients.

Deprivation has been linked with an increase in emergency department attendance and emergency admission rates [8,9]. The quality and outcomes framework (QOF) is a pay for performance scheme used in UK primary care that rewards practices according to their achievement of indicators, including for the management of common chronic conditions; we included total and clinical achievement scores because good primary care may be expected to use of unscheduled care [22]. Access to, and continuity of, care in general practice are also associated with use of secondary care [8,9], and therefore we used data from the annual general practitioner patient survey undertaken as part of the QOF. We used the proportion of patients able to see a GP at their practice within 2 working days as a measure of access, and the proportion able to consult their preferred GP as a measure of relational continuity. Evidence from a study using simulated patients supports the validity of the patient survey [23].

We took the proportion of patients over 65, the gender breakdown of the patients, the headcount number of GPs per 1000 patients, and the deprivation of the area using the Index of Multiple Deprivation from the Health and Social Care Information Centre portal [20]. We also used the prevalence, expected prevalence and prevalence gap for coronary heart disease, stroke and hypertension. We acquired data on the ethnicity of the patients in each practice, in terms of white/non-white, from the Public Health Observatories database; these data were from the years 2005–2007, the most recent data available [24].

### Analysis

We undertook a descriptive analysis of the seven groups of practices, using univariate linear regression to test for trend relationships between variables and the proportion of admissions that were emergency. We then undertook a multivariate analysis using forward stepwise linear regression to identify predictors of the proportion of hospital admissions that were emergency [25]. The selection of variables for inclusion in the regression was designed to account for key patient population characteristics (deprivation, proportion aged 65 or over, proportion who were white, proportion who were male), and characteristics of practices that reflect the systematic management of patients with chronic conditions. These therefore included the proportion of clinical QOF scores achieved, the numbers of outpatient referrals per 10,000 registered patients, the numbers of patients recorded with a diagnosis of hypertension as an indication of success in detecting this chronic condition, and the proportion of patients able to consult their preferred GP. We also included practice list size as larger practices may have greater administrative and nursing capacity available to them. We hypothesised that population characteristics and features of practices reflecting systematic management of chronic conditions would be associated with a lower proportion of admissions being emergency. SPSS version 20 was used to undertake the analyses.

### Ethical approval

No ethical approval was required because this study used publically available data and required no patient contact.

## Results

Of the 233 practices, data on secondary care use were not available for four practices, and these were excluded. The proportion of admissions that were emergency, per practice, ranged from 31% to 90%, mean 44.9% (standard deviation 8.7%). The mean numbers of outpatient appointments, elective admissions, emergency admissions and emergency department attendances per 1000 registered patients in each of the seven groups of practices are shown in Table 1. The population characteristics are shown in Table 2, and practice characteristics in Table 3. Practices with higher proportions of admissions that were emergency tended to have fewer older patients, higher proportions of male patients, fewer white patients, greater deprivation, and lower recorded prevalence of coronary heart disease, hypertension and stroke, as well as smaller list sizes, lower total and clinical QOF points achieved, and bigger gaps between expected and recorded coronary heart disease and stroke prevalence.

**Table 1:** The proportions of total admissions that were emergency, categorised into 7 groups of practices (means and standard deviations [SD])

**Category**	**Mean**	**1**	**2**	**3**	**4**	**5**	**6**	**7**		
**Percentage of admissions that were emergency**		**<36%**	**36-40%**	**41-45%**	**46-50%**	**51-55%**	**56-60%**	**>60%**		
**n**	229	22	51	60	35	30	18	13	beta	p
**Emergency Admissions**/**1000 Patients**	88.6 (84.6-92.6)	64.9 (60.2-69.5)	77.3 (74.2-80.3)	85.8 (82.9-88.8)	87.4 (80.3-94.5)	99.7 (92.9-106.6)	110.1 (98.9-121.2)	134.0 (76.4-191.6)	0.002	<0.001
**Elective Admissions**/**1000 Patients**	108 (104.9-111.9)	130.4 (120.9-140.0)	128.0 (123.0-132.9)	117.8 (114.1-121.5)	95.5 (88.0-103.0)	90.87 (84.5-97.2)	82.4 (73.2-91.6)	62.9 (50.5-75.2)	0.002	<0.001
**OPD Appointments**/**10000 Patients**	6704.55 (6550.1-6858.9)	6238.2 (5764.8-6711.5)	6626.3 (6303.6-6949.0)	7183.8 (6935.3-7432.4)	650.08 (6010.9-7005.1)	686.1 (6512.1-7209.2)	6580.7 (5986.6-7175.7)	5927.7 (5034.8-6820.6)	−0.002	0.496
**ED attendance**/**10000 Patients**	2115.2 (2001.5-2229.9)	1822.3 (1665.1-1979.4)	2069.0 (1924.7-2213.3)	1971.8 (1816.9-2126.7)	1881.4 (1667.4-2095.4)	2262.7 (2124.7-2400.6)	2448.7 (2179.1.9-2716.4)	3282.3 (1607.4-4957.2)	0.053	<0.001

**Table 2:** Patient population characteristics of practices in 7 categories for use of hospital services

**Category**		**1**	**2**	**3**	**4**	**5**	**6**	**7**		
**Percentage of admissions that were emergency**		**<36%**	**36-40%**	**41-45%**	**46-50%**	**51-55%**	**56-60%**	**>60%**		
**n**	229	22	51	60	35	30	18	13	beta	p
**Proportion of Patients aged** ≥ **65**	0.147 (0.140-0.153)	0.160 (0.144-0.175)	0.161 (0.153-0.170)	0.166 (0.157-0.176)	0.142 (0.125-0.160)	0.129 (0.110-0.147)	0.110 (0.095-0.125)	0.081 (0.042-0.120)	−0.860	<0.001
**Proportion of male Patients**	0.509 (0.505-0.514)	0.501 (0.497-0.505)	0.503 (0.499-0.507)	0.500 (0.497-0.504)	0.510 (0.502-0.518)	0.510 (0.503-0.518)	0.523 (0.506-0.541)	0.565 (0.500-0.630)	1.242	<0.001
**White Ethnicity Proportion**	0.801 (0.770-0.832)	0.951 (0.938-0.963)	0.918 (0.897-0.938)	0.890 (0.870-0.910)	0.728 (0.636-0.820)	0.598 (0.480-0.717)	0.544 (0.392-0.697)	0.664 (0.475-0.852)	−0.185	<0.001
**Index of Multiple Deprivation**	19.87 (18.48-21.26)	11.96 (9.68-14.23)	14.91 (13.09-16.73)	14.64 (12.98-16.30)	20.32 (17.35-23.29)	27.86 (24.66-31.07)	35.52 (31.08-39.96)	34.34 (29.96-40.73)	0.006	<0.001
**Coronary Heart Disease Expected Prevalence Proportion**	0.043 (0.041-0.044)	0.041 (0.037-0.045)	0.043 (0.041-0.045)	0.043 (0.042-0.046)	0.042 (0.038-0.047)	0.044 (0.040-0.048)	0.044 (0.039-0.049)	0.035 (0.022-0.047)	−0.382	0.493
**Hypertension Expected Prevalence Proportion**	0.238 (0.234-0.243)	0.245 (0.232-0.258)	0.250 (0.244-0.256)	0.249 (0.243-0.256)	0.234 (0.217-0.251)	0.226 (0.211-0.241)	0.214 (0.202-0.226)	0.198 (0.160-0.235)	−0.811	<0.001
**Stroke Expected Prevalence**	0.019 (0.018-0.019)	0.018 (0.017-0.020)	0.019 (0.018-0.020)	0.020 (0.019-0.020)	0.018 (0.016-0.020)	0.018 (0.016-0.020)	0.018 (0.016-0.020)	0.015 (0.011-0.020)	−3.123	0.015

Means (95% CIs) N = 229.

**Table 3:** Practice characteristics of practices in 7 categories for use of hospital services

**Category**	**Mean**	**1**	**2**	**3**	**4**	**5**	**6**	**7**		
**Percentage of admissions that were emergency**		**<36%**	**36-40%**	**41-45%**	**46-50%**	**51-55%**	**56-60%**	**>60%**		
**n**	229	22	51	60	35	30	18	13	beta	p
**Practice List Size**	7618 (6986–8250)	7584 (5809–9359)	9563 (8121–11005)	9078 (7723–10433)	6939 (5571–8306)	5429 (4137–6721)	5503 (3680–7326)	3264 (889.6-5639)	−6.213E0.006	<0.001
**Coronary Heart Disease Prevalence Proportion**	0.031 (0.029-0.032)	0.030 (0.033-0.032)	0.032 (0.031-0.034)	0.033 (0.031-0.035)	0.031 (0.027-0.034)	0.029 (0.026-0.032)	0.027 (0.024-0.031)	0.021 (0.014-0.027)	−3.691	<0.001
**Coronary Heart Disease Prevalence Gap**	0.012 (0.011-0.013)	0.010 (0.008-0.013)	0.011 (0.009-0.012)	0.011 (0.009-0.012)	0.012 (0.010-0.014)	0.015 (0.012-0.018)	0.017 (0.013-0.020)	0.013 (0.008-0.018)	3.348	<0.001
**Hypertension Prevalence Proportion**	0.134 (0.129-0.138)	0.145 (0.134-0.156)	0.144 (0.137-0.150)	0.139 (0.133-0.145)	0.137 (0.123-0.150)	0.126 (0.0.112-0.139)	0.114 (0.099-0.130)	0.0871 (0.059-0.115)	−1.061	<0.001
**Hypertension Prevalence Gap**	0.104 (0.101-0.107)	0.100 (0.071-0.131)	0.106 (0.101-0.011)	0.110 (0.076-0.153)	0.097 (0.055-0.139)	0.100 (0.090-0.105)	0.100 (0.090-0.110)	0.104 (0.090-0.111)	−0.332	0.186
**Stroke Prevalence**	0.015 (0.014-0.015)	0.015 (0.014-0.017)	0.016 (0.015-0.017)	0.017 (0.016-0.017)	0.015 (0.013-0.017)	0.013 (0.012-0.015)	0.013 (0.010-0.015)	0.008 (0.005-0.011)	−7.939	<0.001
**Stroke Prevalence Gap**	0.004 (0.003-0.004)	0.003 (0.001-0.005)	0.003 (0.002-0.004)	0.003 (0.002-0.004)	0.003 (0.002-0.004)	0.005 (0.003-0.006)	0.005 (0.003-0.007)	0.007 (0.005-0.010)	6.628	<0.001
**Total Quality Outcomes Framework Proportion**	0.948 (0.943-0.954)	0.971 (0.964-0.979)	0.954 (0.946-0.962)	0.953 (0.945-0.961)	0.952 (0.931-0.969)	0.948 (0.933-0.962)	0.919 (0.902-0.937)	0.897 (0.847-0.946)	−0.730	<0.001
**Clinical Quality Outcomes Framework Proportion**	0.971 (0.965-0.976)	0.991 (0.988-0.995)	0.974 (0.965-0.984)	0.977 (0.970-0.984)	0.972 (0.954-0.989)	0.972 (0.957-0.987)	0.989 (0.926-0.971)	0.920 (0.873-0.967)	−0.681	<0.001
**Proportion seen by GP within 2 Working Days**	0.819 (0.806-0.832)	0.842 (0.801-0.883)	0.814 (0.788-0.840)	0.826 (0.801-0.850)	0.846 (0.811-0.881)	0.788 (0.747-0.829)	0.776 (0.722-0.830)	0.833 (0.750-0.916)	−0.068	0.228
**Proportion able to see their Preferred Doctor**	0.686 (0.666-0.708)	0.772 (0.716-0.827)	0.702 (0.662-0.742)	0.703 (0.658-0.747)	0.673 (0.614-0.732)	0.630 (0.556-0.703)	0.620 (0.522-0.718)	0.644 (0.515-0.772)	−0,116	<0.001
**GPs/1000 (mean)**	0.737 (0.361-1.375)	0.720 (0.360-2.720)	0.587 (0.406-0.844)	0.632 (0.369-0.934)	0.605 (0.282-1.205)	0.787 (0.285-3.250)	0.649 (0.244-1.009)	1.220 (0.309-2.635)	0.045	<0.001

Means (95% CIs). N = 229.

For the multivariate analysis, all data were available for 221 practices. The analysis identified patient population characteristics as predictors of the proportion of admissions that were emergency, with greater deprivation, more male patients and fewer white patients being associated with a higher proportion (Table 4). These were the most powerful predictors, but practice characteristics also predicted the proportion of emergency admissions. Higher practice list size, and higher recorded prevalence of hypertension were associated with a lower proportion of emergency admissions, and higher outpatient referrals were association with a higher proportion. However, the proportion of clinical QOF scores achieved and the proportion of patients able to consult their preferred GP did not predict the proportion of admissions that were emergency. The regression model explained 64% of the variation in the proportion of admissions that were emergency.

**Table 4:** **Predictors of the proportion of admissions that were emergency** (**linear regression**, **n** = **221**)

**Variable**	**Beta**	**SE Beta**	**Sig**	**Adjusted R Sq**
Constant	0.026	0.075		
Deprivation index	0.004	0.000	<0.001	0.473
Proportion of patients who were male	0.720	0.116	<0.001	0.572
Outpatient appointments per 10,000 patients	0.002	0.000	<0.001	0.590
Proportion of white patients	−0.001	0.000	<0.001	0.616
Recorded hypertension prevalence	−0.461	0.129	<0.001	0.629
Practice list size/100	−0.002	0.001	0.001	0.644
Proportion aged 65 or over	0.063	0.458	0.648	
Proportion of total clinical QOF points obtained	−0.053	−1.148	0.253	
Proportion of patients able to consult their preferred GP	−0.039	−0.845	0.399	

Beta indicates the amount of change for one unit change of the independent variable, controlling for the other independent variables. Thus, an increase of 1% in the proportion of the practice population who are male is associated with a 0.7% increase in the proportion of admissions that are emergency, whilst a 1% increase in recorded prevalence of hypertension is associated with a 0.46% decrease in the proportion of admissions that are emergency.

## Discussion

### Summary of the main findings

In this study we set out to characterise general practices by the proportion of admissions that were emergency. Wide variation in the proportion of admissions that were emergency was found, and it was possible to allocate practices to different groups according to the proportion. There were differences in the populations of practices in these groups, and some differences in the characteristics of practices.

Whilst population characteristics did predict the proportion of admissions that were emergency, practice characteristics indicative of systematic management were also important. Better detection of hypertension was associated with lower proportions of admissions being emergency. Higher detection of hypertension has been shown to be associated with lower coronary heart disease mortality [26], which may be explained by higher mortality among people who have hypertension that is not controlled and the finding that 16% of men and 11% of women had untreated hypertension in the Health Survey for England in 2012 [27]. Larger practices may have greater organisational and management capacity that are associated with lower proportions of admissions being emergency. The positive association of outpatient referrals to the proportion of admissions that were emergency suggests that a high use of outpatient referral may have little impact in containing emergency admissions. These findings highlight the importance of practice populations in driving the use of secondary care, but also the interaction between practice populations and the ability of practices to fully meet the needs for care of those populations. If practices are unable to meet the needs of their populations, greater use of secondary care services is more likely. The failure of QOF clinical achievement scores to predict the proportion of admissions that were emergency may be partly explained by the fact that not all admissions occur in people who have a chronic condition, but in those that do, the QOF variable relates to a large number of different chronic conditions. It remains possible that more effective care for specific conditions does reduce rates of emergency admissions for exacerbations of those conditions [7].

### Strengths and limitations of the study

Our study has used data about practices in three counties of England, and the findings may not be fully generalizable to practices in other parts of England, or other countries. We used available data on general practices, and some of these data had limitations. The response rates to the general practitioner patient survey are low, although the validity of the survey has been demonstrated in other research [23]. The data on the ethnicity of practice populations was estimated from hospital in-patient figures and may not fully reflect the population in the community, but alternative sources of more accurate data were not available. Moreover, we were unable to account for variations in the structure and organization of secondary care services that may affect hospital use, or geographical factors such as distance of patients from their local hospital. Also, some potentially relevant data were not available, for example, the numbers of nurses per practice.

Our study is observational and can only highlight associations. Empirical studies that test interventions are required to confirm the effect on emergency admissions of improved management of chronic disease by practice teams.

### Implications

Containing the use of secondary care services is a priority for the health systems in many countries. In England, the 2013/14 version of the QOF incorporates indicators for quality and productivity that include peer review of outpatient referrals, emergency admissions, and emergency department attendances [28]. The NHS outcomes framework is used to provide a national level overview of how well the service is performing and be a catalyst for driving improvement in outcomes, and includes data on emergency admissions that should not usually require hospital admission [29]. In monitoring use of secondary care services as an outcome of primary care, our study highlights the importance not only of understanding the role of population characteristics in determining levels of use, but also of appreciating the ability of practices to meet the demands for care that face them. The level of resources, and the way in which available resources are used, are likely to be key factors in determining whether a practice is able to meet the health care needs of its patients.

In rising to the challenge of an ageing and multi-morbid population, reforms of primary care are being proposed in England. These include the creation of federations or other forms of non-commercial or commercial groupings of practices that are able to share innovations and improve efficiency [30,31]. If practices themselves, or federations of practices, are to prevent the rising tide of chronic disease from swamping hospital services, they need to target resources to where they are needed, and adopt effective innovations quickly. A typology based on the proportion of admissions that are emergency could help individual practices, federations or other organizations that oversee general practices improve their understanding of practice populations and the ability of practices to care for them. Our typology does not reveal how some practices with similar populations and resources make less use of secondary care than others, and this suggests opportunities for practices to learn from each other. It also suggests that further research should seek to investigate how practices identify and respond to the needs of their populations. Our study is also an example of how the increasing amount of publicly available data about general practice may be used to better understand how processes of care can influence outcomes such as hospital admissions. As even more data become available, practices and clinical commissioning groups will be increasingly able to compare performance and identify factors influencing admission rates.

## Conclusions

The characteristics of practice populations are important in determining use of secondary care services. Characteristics of practices also influence use of secondary care services, and in particular, the extent to which practices are able to meet the needs of their populations is important. In order to improve the outcomes of primary care such as use of secondary care, processes are required to describe the small populations of individual practices, and to target resources and support to practices and their populations that need them.

## Competing interests

The authors’ declare that they have no competing interests.

## Authors’ contribution

CW and RB jointly conceived the study. CW collected the data, and prepared a first draft of the paper. RB revised the paper. Both authors read and approved the final manuscript.

## Pre-publication history

The pre-publication history for this paper can be accessed here:

http://www.biomedcentral.com/1471-2296/15/101/prepub
